# AN OBJECTIVE MEASURE OF STRESS REACTIVITY/RESILIENCE IN BLACK WOMEN DEMENTIA FAMILY CAREGIVERS

**DOI:** 10.1093/geroni/igae098.3123

**Published:** 2024-12-31

**Authors:** Kathy Wright, Karen Moss, Nathan Helsabeck, Ingrid Richards Adams, Karen Rose, Maryanna D Klatt

**Affiliations:** The Ohio State University, Columbus, Ohio, United States; The Ohio State University, Columbus, Ohio, United States; The Ohio State University, Columbus, Ohio, United States; The Ohio State University, Columbus, Ohio, United States; The Ohio State University, Columbus, Ohio, United States; The Ohio State University, Columbus, Ohio, United States

## Abstract

Real-time objective measures of stress are crucial for identifying mechanisms applicable to health behavior change research for dementia caregivers. We explore the Pittsburgh Stress Battery (PSB) in a non-laboratory setting (R21AG077069) as an objective measure of stress reactivity/resilience. The PSB was administered to 16 Black American women caregivers with hypertension (40-65 years old). PSB includes three challenging tasks (Stroop, Mental Math, and Mirror Tracing). An automatic cuff measured systolic blood pressure (SBP), diastolic blood pressure (DBP) and heart rate (HR) at three intervals during the 20-minute battery followed by a 30-minute recovery. Average participant age was 62.4 (SD= 6.1). Ten assessments were completed in an office and six at home. One Stroop and three Mental Math were not completed due to computer/internet issues or refusal. Mean SBP, DPB, and HR at baseline were 128.9 mmHg, 81.6 mmHg, and 71.3 (beats per minute). The difference from baseline in SBP was 10.5 mmHg (SD= 8.3) for Stroop, Mental Math -.94 mm Hg (SD= -0.6), and Mirror Tracing 1.6 mmHg (SD= 9.4). For DBP, Stroop was 5.0 mm Hg (SD=4.6), Mental Math 0.2 mm Hg (SD= 3.2), and Mirror Tracing 0.3 mm Hg (SD= 7.0). HR difference from baseline was 3.8 (SD= 0.5), Mental Math -4.24 (SD= 8.2), and Mirror test -0.75 (SD= -3.4). Findings indicate that PSB elicits SBP/DBP/HR stress reactivity/resilience. PSB was portable for use in community settings. Study results will help guide interventions for behavior change, aiming to improve health outcomes in Black Americans.

